# Climate change‐driven expansion of goosegrass highlights risks to global food production

**DOI:** 10.1002/ps.70731

**Published:** 2026-03-15

**Authors:** Thiago Deomar Ludwig, Ednaldo Alexandre Borgato, Luis Antonio de Avila, Laura Montipó Roncaglia, Maria Imaculada Zucchi

**Affiliations:** ^1^ Departamento de Genética, Escola Superior de Agricultura ‘Luiz de Queiroz’ Universidade de São Paulo (ESALQ/USP) Piracicaba Brazil; ^2^ West Florida Research and Education Center (WFREC) – Institute of Food and Agricultural Sciences (UF/IFAS) University of Florida Jay FL USA; ^3^ Department of Plant and Soil Sciences Mississippi State University Starkville MS USA; ^4^ Instituto de Biologia Universidade Estadual de Campinas (UNICAMP) Campinas Brazil; ^5^ Agência Paulista de Tecnologia dos Agronegócios (APTA) Piracicaba Brazil

**Keywords:** ecological niche modeling, *Eleusine indica*, global food security, integrated weed management, range expansion, weed

## Abstract

**BACKGROUND:**

Climate change and the spread of herbicide‐resistant weeds pose increasing risks to global food security. *Eleusine indica* (goosegrass) is a globally invasive species characterized by broad geographic distribution, high ecological plasticity, and multiple resistance mechanisms. This study evaluated the current and future climatic suitability of *E. indica* at a global scale under climate change scenarios.

**RESULTS:**

Ensemble ecological niche models calibrated with georeferenced occurrence records and climatic predictors showed excellent performance (area under the receiver operating characteristic curve (AUC) = 0.999; true skill statistic (TSS)/kappa = 0.980), with low omission rates and high spatial agreement among algorithms (Bioclim, Random Forest, Support Vector Machines, and Maxent). Current projections indicate widespread suitability across tropical and subtropical regions, particularly in South America, Africa, and Southeast Asia. Future projections under SSP245 and SSP585 for 2050 and 2090 suggest a gradual expansion toward higher latitudes, with increased suitability in temperate regions such as the US Corn Belt, the Mediterranean Basin, and East Asia, especially under SSP585 by 2090. Overlap analyses identified substantial vulnerability in major soybean‐producing regions, including Brazil, Argentina, the United States, and Southeast Asia.

**CONCLUSION:**

Climate change is expected to facilitate the poleward expansion of *E. indica*, increasing invasion risk in key agricultural regions while maintaining broad climatic stability across its current range. These findings emphasize the need for early detection, continuous monitoring, and integrated weed management strategies to mitigate long‐term agronomic and food security risks posed by this globally invasive species. © 2026 The Author(s). *Pest Management Science* published by John Wiley & Sons Ltd on behalf of Society of Chemical Industry.

## Graphical abstract

Climate change is expanding the climatic suitability of *Eleusine indica* into temperate agricultural regions, increasing its overlap with major soybean and maize production areas. These findings highlight a growing global threat to food security and the need for proactive weed management strategies.

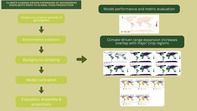

## 1 INTRODUCTION

Global food security is increasingly threatened by the growing demand for agricultural production and the slowdown in productivity gains, particularly in developing countries.^1^ Climate change, driven by global warming over the past 150 years, exacerbates this challenge by intensifying extreme events and altering climate patterns that compromise crop yields and food supply.^2^, ^3^

Agriculture is among the sectors most vulnerable to climate change, being directly affected by droughts, floods, and unpredictable fluctuations in temperature and precipitation, factors that reduce crop yields and heighten uncertainties regarding global food security.^4^, ^5^, ^6^, ^7^ The model of agricultural intensification consolidated over the past two centuries, characterized by the expansion of cultivated areas, the predominance of monocultures, and the intensive use of chemical inputs, enabled significant productivity gains but also increased the dependence on high‐impact environmental practices.^8^

Since the mid‐20th century, chemical weed control has become a cornerstone of this production system, supporting the spread of high‐yield cultivars.^9^, ^10^ Although this strategy initially boosted production, its intensive use created conditions that favored the evolution of herbicide‐resistant weeds, now recognized as one of the greatest threats to the sustainability of modern agriculture, as they directly reduce crop yields, compromise the effectiveness of chemical management, and increase production costs.

In parallel, the convergence of climatic suitability and anthropogenic disturbance alters the spatial distribution of invasive grass species across broad geographic extents. At regional scales, climate change and land‐use intensification reshape invasion risk, leading to climate‐driven shifts in habitat suitability that define areas of habitat loss, persistence, and localized gain under contrasting emission scenarios.^11^, ^12^

Within this context, *Eleusine indica* (L.) Gaertn. (goosegrass) stands out for its remarkable ecological plasticity and the evolution of herbicide resistance, posing a growing threat to agriculture under future climate scenarios.^1^, ^13^, ^14^ Among the most economically relevant weeds, *E. indica* is a grass species widely distributed across tropical and subtropical regions. This invasive species infests cereals, legumes, and vegetables, compromising the yield of essential crops such as soybean and maize (corn).^15^

Since the first report of resistance in *E. indica*, its frequency has increased significantly, largely driven by the intensive use of herbicides, particularly glyphosate.^16^ This adaptive success is attributed to its high genetic variability and the coexistence of multiple resistance mechanisms, including gene amplification, target‐site mutations, regulatory alterations, and enhanced metabolic detoxification of herbicides.^17^, ^18^ Recent studies documenting glyphosate‐resistant *E. indica* populations in both Asia and Europe provide clear evidence that resistance evolution has accompanied the species' expansion beyond its native range, reinforcing its capacity to establish and spread across diverse agroclimatic regions and agricultural systems.^19^, ^20^

Beyond intrinsic biological and evolutionary traits, the distribution and spread of *E. indica* are strongly shaped by extrinsic environmental factors operating across multiple spatial scales. At broad spatial extents, climatic conditions, particularly temperature and precipitation regimes and their seasonality, delimit the potential geographic distribution of the species.^21^, ^22^, ^23^ At finer local scales, environmental heterogeneity associated with topography, microclimatic variation, and landscape structure further modulates habitat suitability, influencing establishment success and local population persistence. The interplay between large‐scale climatic constraints and small‐scale environmental conditioning factors is critical for characterizing invasion processes and anticipating future range dynamics under climate change.^24^

In this scenario, an ecological niche model (ENM) emerges as an essential tool to understand the current and potential distribution of *E. indica*, particularly under climate change scenarios. This approach integrates environmental and biological information to predict areas susceptible to the occurrence and expansion of the species, providing valuable insights for the planning of more effective and sustainable management strategies.^25^ By identifying high‐risk regions and assessing how climatic factors may influence its adaptability, ENM contributes to anticipating agricultural impacts and guiding preventive actions that help preserve productivity and food security.

Thus, this study aims to evaluate the current and future distribution of *E. indica* through an ENM, integrating occurrence data and climatic variables under global change scenarios (SSP245 and SSP585 for 2050 and 2090). The spatial projection of climatic suitability makes it possible to identify areas at risk of species expansion and regions potentially affected by the intensification of herbicide resistance. In addition, soybean distribution was modeled, and binary maps were integrated with *E. indica* projections to identify overlapping areas, highlighting critical regions for agricultural management under future climate scenarios. These results provide support for agricultural planning and the development of regional sustainable management strategies, contributing to mitigating the impacts of this species on agricultural systems and global food security.

## 2 MATERIALS AND METHODS

### 2.1 Data collection and filtering

Species occurrence records were obtained from digital herbarium repositories through the *spocc* package in R (https://github.com/ropensci/spocc), which consolidates information from multiple databases such as the Global Biodiversity Information Facility (GBIF, https://www.gbif.org/), the New York Botanical Garden (https://www.nybg.org/), and SpeciesLink (https://specieslink.net/).

To ensure data quality and spatial accuracy, the dataset was subjected to a stringent cleaning process with the *CoordinateCleaner* package.^26^ The following filters were applied: (i) removal of entries without latitude/longitude, duplicated coordinates, or with values equal to zero; (ii) spatial checks excluding records located within 1 km of national capitals, 10 km from country or municipality centroids, and 100 m from major biodiversity institutions (e.g., GBIF headquarters). Points falling in oceanic or urban zones were also discarded; (iii) identification of extreme geographic outliers, where the five most discrepant points were excluded using the quantile method; (iv) verification of taxonomic identity and geographic reference, with inconsistent records manually reviewed and excluded. Additionally, records dated before 1980 were excluded due to potential inaccuracies in georeferencing practices during that period.^27^ Entries with missing, null, or incorrect year information were also removed from the dataset.

### 2.2 Climatic data inputs for modeling

To characterize current climate conditions, we used 19 bioclimatic predictors (BIO1–BIO19) available from the WorldClim 2.1 database were used, which represent the baseline period 1970–2000 at a spatial resolution of 5 arcminutes (Supporting Information Table S1). These layers describe central dimensions of climate, including long‐term averages of temperature and precipitation, indicators of intra‐annual variability, and measures of environmental extremes such as the coldest monthly temperature or rainfall during the driest quarter.^28^, ^29^

Future climate layers were derived from simulations of two global circulation models (GCMs), MPI‐ESM1‐2‐HR and EC‐Earth3‐Veg.^30^, ^31^ Projections were generated under two shared socioeconomic pathways (SSPs), which represent alternative trajectories of socioeconomic development, greenhouse gas emissions, and climate policies. We considered SSP245 and SSP585, corresponding to an intermediate stabilization pathway and a high‐emission, fossil fuel‐driven pathway, respectively. Under SSP245, radiative forcing reaches approximately 4.5 W m^−2^ by 2100, whereas SSP585 reflects continued high greenhouse gas emissions, with radiative forcing of approximately 8.5 W m^−2^ and atmospheric carbon dioxide (CO_2_) concentrations exceeding 900 ppm by the end of the century.^32^ To examine potential temporal changes in species distribution, two future projection windows were considered: 2050, representing mean climatic conditions for the period 2041–2060, and 2090, corresponding to average conditions for 2081–2100.

### 2.3 Ecological niche modeling

After selecting species occurrence records with the *CoordinateCleaner* package, the ENM was built using ENMTML.^33^ To minimize spatial sampling bias, we applied a spatial thinning procedure that excluded records located within a minimum distance equal to twice the grid‐cell resolution of the environmental layers. This ensured that no two retained occurrences were closer than this threshold, reducing redundancy in densely sampled areas and preventing model overfitting.

To address multicollinearity among predictors, a principal component analysis (PCA) was performed, and six principal components cumulatively explaining 96.8% of the total variance were retained for model calibration (Table S2). The contribution of each original bioclimatic variable to the retained components (PCA loadings) is detailed in Table S3.^34^

To capture different perspectives of species–environment relationships, we applied algorithms representing three major modeling strategies: a presence‐only approach using Bioclim^35^; presence–absence methods represented by Random Forest^36^ and Support Vector Machines (SVM)^37^; and a presence–background framework implemented with Maxent v3.4.1.^38^

Pseudo‐absences were generated with the env_const method, restricting background selection to environmentally unsuitable areas predicted by Bioclim, thus limiting bias. Each algorithm was evaluated using a bootstrap procedure (70% training, 30% testing, repeated ten times).

The ensemble was computed using the mean method (‘MEAN’) to combine predictions. Specifically, ensemble suitability maps were generated by calculating the pixel‐wise arithmetic mean across the predictions of all individual algorithms (Bioclim, Maxent, Random Forest and SVM). For each grid cell, suitability values predicted by each algorithm were averaged, resulting in a continuous consensus surface representing the central tendency across models.

No performance‐based weighting was applied, as all models met the predefined evaluation thresholds (area under the receiver operating characteristic curve (AUC) > 0.75 and true skill statistic (TSS) > 0.7) and exhibited consistently high and comparable predictive performance under the same validation framework.^39^ Therefore, each algorithm contributed equally to the final ensemble prediction. This unweighted averaging approach reduces algorithm‐specific uncertainty and avoids over‐reliance on any single modeling technique, yielding a more stable and ecologically robust suitability map, particularly appropriate for global‐scale projections.

Suitability predictions were maintained as continuous maps to avoid distortions associated with arbitrary threshold selection; however, for descriptive purposes, binary maps were generated using the maximum true skill statistic (MAX_TSS) criterion. Spatial autocorrelation in model residuals was assessed using Moran's *I* to detect potential spatial structure not captured by the models. To evaluate extrapolation risk under future climate scenarios, environmental novelty was examined using both multivariate environmental similarity surface (MESS) and mobility‐oriented parity (MOP) analyses, allowing the identification of regions characterized by non‐analog climatic conditions and strict extrapolation beyond the calibration domain.^28^, ^40^

Additionally, binary suitability maps were generated for *Glycine max* (soybean) and *Zea mays* (maize) using the same modeling framework, allowing comparative analyses of potential overlap between major crop cultivation zones and suitable habitats for *E. indica* under current and future climatic scenarios.

Maps were generated and visualized in R with *raster*,^41^ ggplot2,^42^ sf^43^ and gridExtra^44^ R packages.

## 3 RESULTS

**Figure 1:**
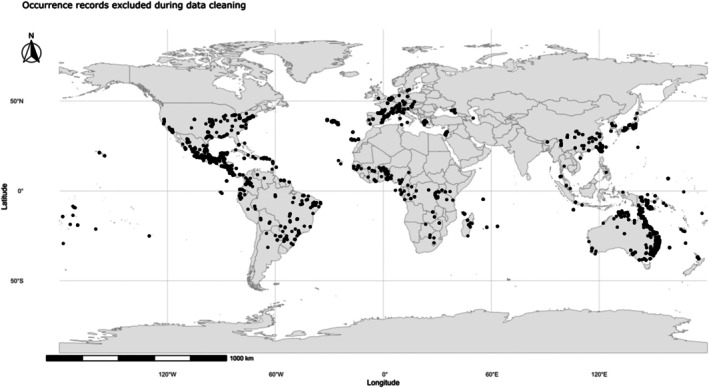
Occurrence records of *Eleusine indica* excluded from modeling after spatial filtering. Black points indicate georeferenced records that were removed during data cleaning due to spatial inconsistencies or redundancy, including duplicated coordinates and points located near centroids, urban areas, or outside terrestrial boundaries. These records correspond to occurrences filtered out prior to ecological niche modeling to ensure spatial accuracy and model reliability.

A total of 11 609 occurrence records of *E. indica* were initially retrieved from online repositories. After the data‐cleaning procedures described earlier, including the removal of duplicates, records with missing or inconsistent coordinates, points located in urban or oceanic areas, and those dated before 1980, 4696 high‐quality georeferenced occurrences were retained for modeling (Fig. 1).

All algorithms showed significant predictive performance, with AUC values above 0.99 for most models and consistently kappa (concordance coefficient) and TSS (> 0.92). Similarity indices (Jaccard = 0.925–0.982; Sorensen = 0.961–0.991) supported the robustness of predictions, while omission rates remained low (< 0.05). The ensemble model combined the outputs of individual algorithms, reaching AUC = 0.999 and TSS/kappa = 0.980; standard deviation ≤ 0.007 across replicates (Table 1).

**Table 1:** Mean (± standard deviation) performance metrics of ecological niche models (ENMs) for *Eleusine indica*

Algorithm	AUC	Kappa	TSS	Jaccard	Sorensen	OR
BIO	0.996 ± 0.001	0.966 ± 0.006	0.966 ± 0.006	0.966 ± 0.006	0.983 ± 0.003	0.031
MXS	0.992 ± 0.002	0.923 ± 0.007	0.923 ± 0.007	0.925 ± 0.007	0.961 ± 0.004	0.046
RDF	0.999 ± 0.001	0.982 ± 0.002	0.982 ± 0.002	0.982 ± 0.002	0.991 ± 0.001	0.012
SVM	0.999 ± 0.001	0.976 ± 0.003	0.976 ± 0.003	0.976 ± 0.003	0.988 ± 0.002	0.012
MEAN	0.999 ± 0.001	0.980 ± 0.003	0.980 ± 0.003	0.981 ± 0.003	0.990 ± 0.001	0.012

*Note*: Performance metrics of ENMs for *E. indica*, obtained through bootstrap partitioning and using the maximum true skill statistic (MAX_TSS). Mean values and standard deviations for each metric are presented across replicates. AUC (area under the receiver operating characteristic curve), kappa (concordance coefficient), and TSS (true skill statistic) indicate the models' discriminative capacity, while Jaccard and Sorensen represent similarity indices quantifying the overlap between predicted presence areas and actual occurrence points. OR (omission rate) reflects the proportion of observed presences incorrectly predicted as absences. Variability across replicates is expressed by the standard deviation of each metric.

The residual analysis indicated moderate spatial autocorrelation (Moran's *I* = 0.65 ± 0.01), suggesting that the distribution of presences was not entirely random but spatially structured (Table 2). The MESS (14.33 ± 0.97) showed that the models were projected mainly onto environmental conditions similar to those used for calibration, reducing the risk of extrapolation to non‐analog climates. These results confirm the stability and reliability of the models regarding spatial independence and environmental similarity across replicates.

**Table 2:** Evaluation of spatial autocorrelation and environmental extrapolation of niche models for *Eleusine indica*

Moran's *I*	MESS
0.65 067 ± 0.01301	1 433 424 ± 0.97 262

*Note*: Values of the spatial autocorrelation index (Moran's *I*) and multivariate environmental similarity surface (MESS) along with their respective standard deviations were used to assess the spatial independence of residuals and the reliability of the models' environmental projections.

**Figure 2:**
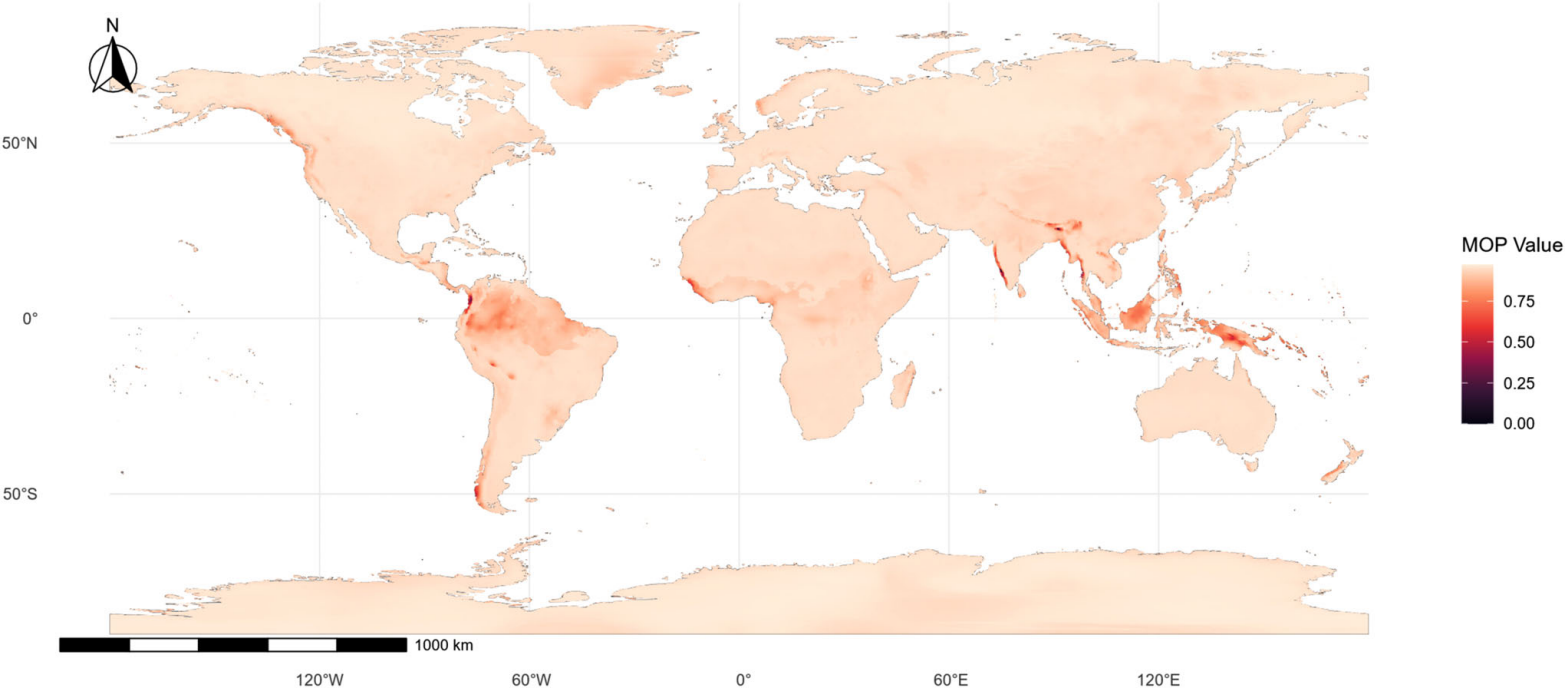
Mobility‐oriented parity (MOP) analysis assessing environmental novelty in future climate projections for *Eleusine indica*. MOP values indicate the degree of environmental similarity between projected conditions and the calibration domain, with lower values representing areas of strict extrapolation where at least one climatic variable falls outside the range observed in the training data. Regions with moderate to high MOP values denote projections supported by environmentally analogous conditions, whereas localized areas with low MOP values highlight zones where suitability predictions should be interpreted with greater caution.

The MOP analysis revealed that, despite the overall environmental similarity indicated by MESS, specific regions are characterized by low environmental parity relative to the calibration domain, indicating areas of strict extrapolation (Fig. 2). Low MOP values were primarily concentrated in parts of Southeast Asia, the Indian subcontinent, northern South America, and localized regions of Central Africa and Oceania. These regions correspond to environmental conditions with low parity relative to the calibration domain and therefore represent areas where projections involve strict extrapolation and should be interpreted with increased caution. In contrast, most global regions exhibited moderate to high MOP values, suggesting that ensemble projections were largely supported by environmentally analogous conditions.

Projections for 2050 indicated moderate changes in suitable area. Under SSP245, the total suitable area reached 71.96 million km^2^, while under SSP585 it increased to 73.08 million km^2^, reflecting a balance between range contraction and expansion (Table 3). By 2090, a more pronounced expansion was projected, particularly under the high‐emission scenario. Under SSP245, the suitable area increased to 73.96 million km^2^, whereas SSP585 projected a substantial increase to 78.91 million km^2^, highlighting the potential for range expansion under high‐emission conditions.

**Table 3:** Changes in climatically suitable areas of *Eleusine indica* under current and future climate scenarios

Climate scenario	Range contraction (current)	Range expansion (SSP)	Stable area (overlap)	Total future suitable area
SSP245 2050	2952036	5387525	66573718	71961243
SSP585 2050	3002177	6557279	66523577	73080856
SSP245 2090	3208847	7641544	66316907	73958451
SSP585 2090	3532483	12919929	65993271	78913200

*Note*: Values are expressed in square kilometers (km^2^). Range contraction and expansion represent areas predicted to become unsuitable or newly suitable, respectively, relative to current climatic conditions. Stable area indicates regions remaining suitable under both current and future scenarios.

**Figure 3:**
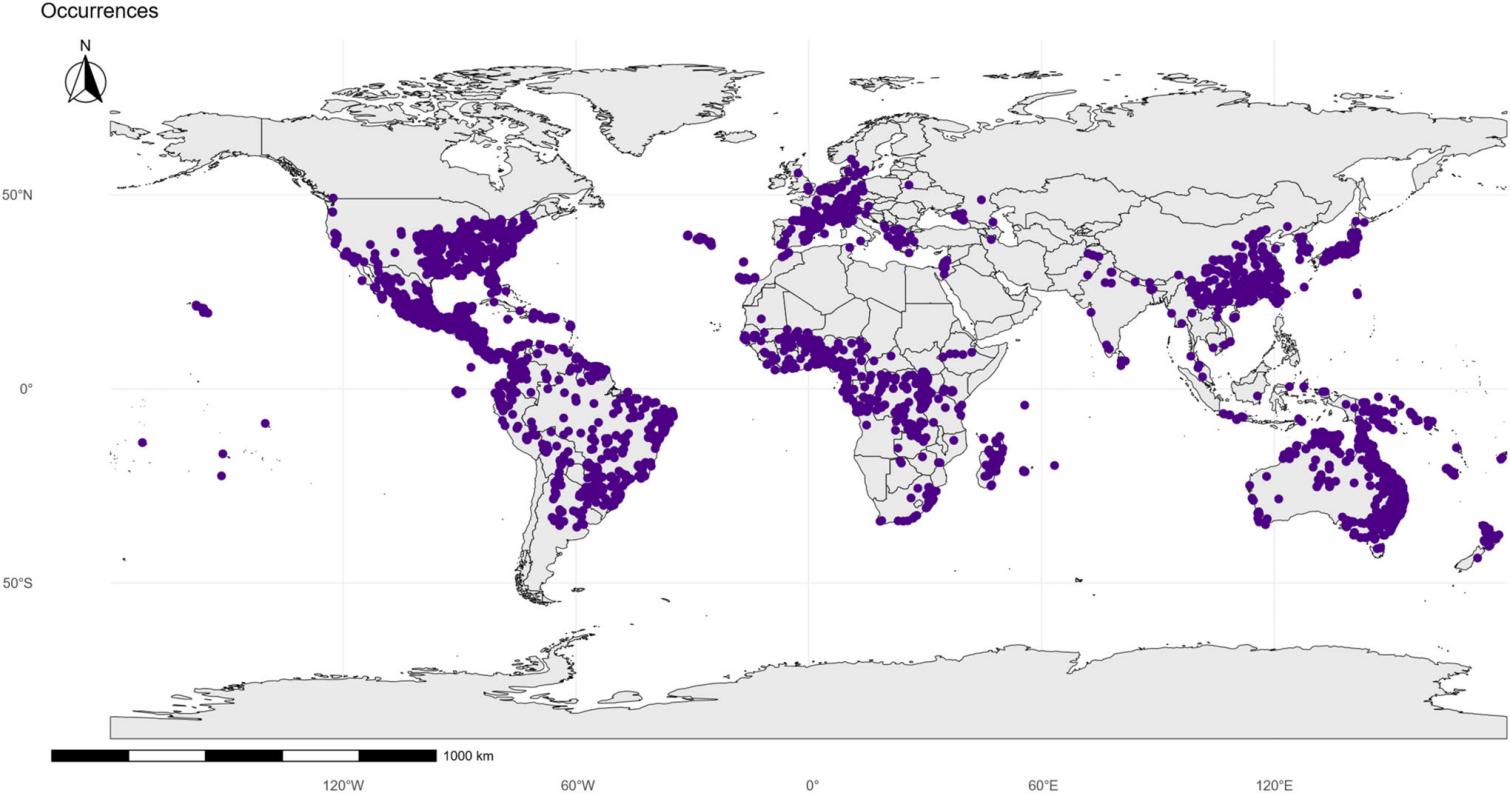
Cleaned occurrence dataset for *Eleusine indica* (goosegrass). Blue points show unique, georeferenced presence records retained after data‐quality screening and spatial filtering (coordinate checks, duplicate removal, environmental/outlier diagnostics, and spatial thinning). This is the final occurrence set used to calibrate and evaluate the ensemble ecological niche model (ENM).

Occurrence records (Fig. 3) confirm the establishment of the species across most tropical and subtropical regions, with a high concentration of their presence in South America, Africa, Southeast Asia, and Oceania. Particularly noteworthy is the strong concentration in Brazil, which constitutes one of the global epicenters of the *E. indica* invasion, accompanied by extensive records in West and East Africa, India, and Southeast Asia. Additional records in North America, southern Europe, and the Mediterranean basin demonstrate the species' ability to extend beyond strictly tropical environments, invading temperate agricultural ecosystems whenever climatic and management conditions are favorable.

**Figure 4:**
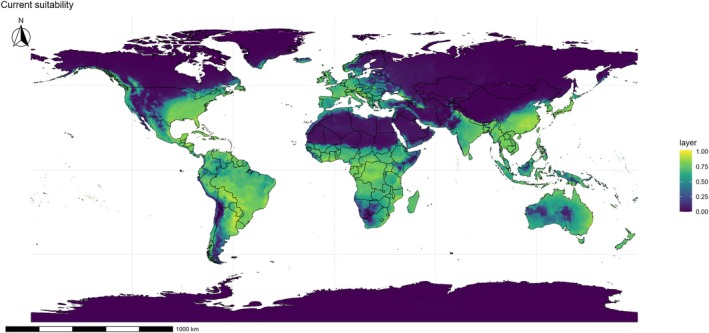
Ensemble ecological niche model (ENM) projection for *Eleusine indica* under current climatic conditions. Warmer colors (yellow–green) represent areas of higher climatic suitability, while cooler colors (blue–purple) indicate low or no suitability. The model reveals extensive areas favorable to the species across South America, Sub‐Saharan Africa, South and Southeast Asia, and Oceania, with additional high‐suitability zones in the southern United States, Mediterranean Europe, and eastern China.

The current ENM (Fig. 4) indicates that the potential distribution of *E. indica* is considerably broader than its presently occupied areas. Vast portions of South America, particularly the Brazilian Cerrado, the Atlantic Forest, and the agricultural frontiers of Argentina and Paraguay, were identified as highly suitable for the species. In North America, extensive areas of the southern United States and even parts of the Corn Belt presented favorable conditions, highlighting the risk of expansion into one of the most strategic regions for global grain production. In Africa, suitability extended across the entire Sahel belt, as well as central and southern regions of the continent, indicating that areas already heavily impacted by weeds remain highly vulnerable to further invasion by *E. indica*.

In Asia, the models revealed high suitability across almost the entire Indian subcontinent and Southeast Asia, including India, Thailand, Vietnam, and southern China regions that represent both biodiversity hotspots and some of the most densely cultivated agricultural lands in the world. Particularly alarming is the suitability projected for eastern China and the Yangtze River basin, an area of strategic importance for rice production, as well as the Indo‐Gangetic Plain, recognized as the breadbasket for more than one billion people. In Oceania, especially Australia, projected suitability was also broad, encompassing both coastal zones and interior agricultural areas, reinforcing reports of *E. indica* as a recurrent problem in cereal and cotton systems. The climatic envelope identified by the models even extends into temperate zones, with emphasis on Mediterranean Europe, where Spain, Italy, and Greece appear as potential expansion areas. This pattern illustrates the remarkable ecological breadth of the species, capable of adapting to environments ranging from humid equatorial conditions to drier subtropical and temperate climates. Such versatility is directly associated with its high genetic variability and the presence of multiple herbicide resistance mechanisms, which together confer adaptive advantages in heterogeneous and rapidly changing environments.

Despite its wide current distribution, there are still extensive areas of suitability that are not yet occupied by *E. indica*. This discrepancy between realized and potential distribution highlights the species' latent invasive capacity. Regions such as India exhibit high suitability but relatively sparse occurrence records, suggesting under‐sampling or ongoing colonization processes. From a management perspective, such areas represent priority zones for monitoring and early detection programs, as preventive measures are much more efficient and economically viable than eradication attempts after populations are established.

The overlap between climatically suitable areas for *E. indica* and the main grain‐producing regions of the world, including soybean and maize areas in Brazil, Argentina, and the United States, as well as rice systems in Asia raises serious concerns for global food security. In these regions, the species not only directly competes with essential crops but also poses increasing challenges due to herbicide resistance. Thus, the maps presented do not merely depict the potential distribution of the species but delineate the geography of future agronomic conflicts and highlight regions where management costs are likely to intensify.

**Figure 5:**
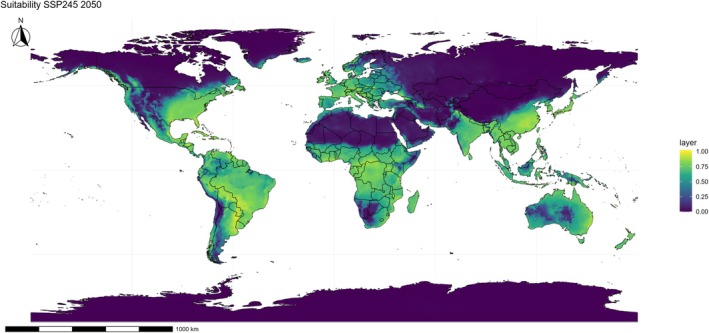
Projected climatic suitability for *Eleusine indica* under the SSP245 scenario for 2050. The ensemble ecological niche model (ENM) shows suitability on a continuous scale, with yellow–green areas indicating favorable conditions and blue–purple areas indicating unsuitable regions. The projection highlights potential future distribution patterns of the species under a moderate‐emission scenario.

**Figure 6:**
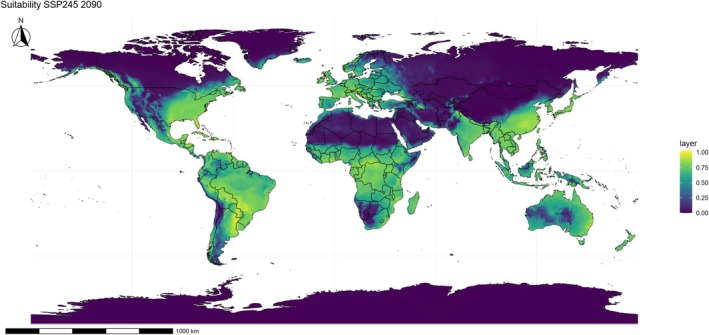
Projected climatic suitability for *Eleusine indica* under the SSP245 scenario in 2090. Suitability is displayed on a continuous color scale, where yellow–green areas indicate favorable conditions and blue–purple areas indicate unsuitable regions. The projection is derived from ensemble ecological niche model (ENM), with political boundaries shown only for spatial reference.

Under the intermediate climate change scenario (SSP245), a clear trend of suitability expansion is observed over the coming decades. By 2050 (Fig. 5), the species encounters even more favorable conditions in strategic agricultural zones, such as the Brazilian Cerrado, the southern United States, the Mediterranean Basin, and South Asia. By 2090 (Fig. 6), suitability intensifies and advances into higher‐latitude temperate regions, particularly the north‐central United States, Eastern Europe, and parts of continental China, suggesting that the species may colonize new agricultural territories that are currently less affected.

**Figure 7:**
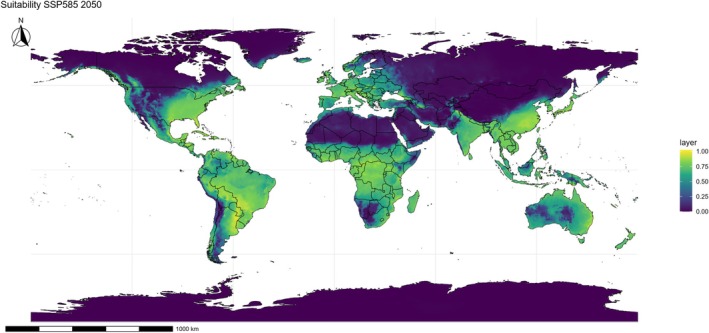
Projected climatic suitability for *Eleusine indica* under the SSP585 scenario in 2050. Suitability is represented on a continuous color scale, with yellow–green areas indicating favorable conditions and blue–purple areas indicating unsuitable regions. The projection is based on ensemble ecological niche model (ENM), with political boundaries shown only for spatial reference.

**Figure 8:**
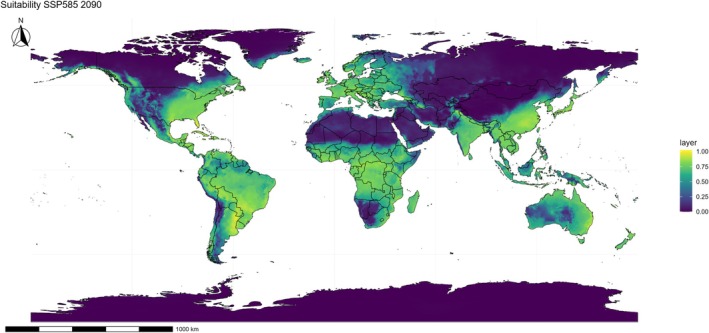
Projected climatic suitability for *Eleusine indica* under the SSP585 scenario in 2090. Suitability is displayed on a continuous color scale, with yellow–green areas indicating favorable conditions and blue–purple areas representing unsuitable regions. The projection is derived from ensemble ecological niche model (ENM), with political boundaries included only for spatial reference.

In the high‐emission scenario (SSP585), projected patterns indicate a markedly more pronounced expansion. By 2050 (Fig. 7), *E. indica* already encounters favorable conditions across most of the Americas, Sub‐Saharan Africa, and Asia, with substantial overlap between suitable areas and the principal grain‐producing regions of Brazil, Argentina, the United States, India, and China. By 2090 (Fig. 8), the potential range reaches its maximum extent, advancing significantly northward and consolidating in temperate regions that were previously marginal, including Canada, Central Europe, and extensive portions of western Russia.

Comparing scenarios, the most pronounced differences between SSP245 and SSP585 occur in climatic frontier zones, such as temperate regions of the Northern Hemisphere and semi‐arid areas of Africa and Asia. While SSP245 maintains a gradual and relatively controlled expansion, SSP585 projects large‐scale invasion, threatening high‐value agricultural areas that until now functioned as climatic barriers to the species' dispersal.

These results reinforce that climate change not only broadens the potential distribution of *E. indica* but also shifts its suitability boundaries toward regions currently less affected. This represents an increasing risk to global food security, as the projected expansion coincides with the main rice, maize, and soybean production areas, raising management costs and intensifying selective pressure for the evolution of new herbicide‐resistant populations.

Despite the robustness of the results, some inherent limitations of ENM must be considered. The projected distribution is based on available occurrence records, which may be subject to sampling gaps and collection bias, particularly in tropical regions and countries with limited monitoring efforts. Moreover, the models were calibrated solely with climatic variables, without considering biotic and anthropogenic factors such as competition with other species, land use, agricultural management intensity, and herbicide rotation, which directly influence the establishment and spread of *E. indica*. Another limitation is that the climate scenarios used represent global projections and do not capture microclimatic or local variations that may alter actual suitability at finer scales. Finally, although the use of multiple algorithms and ensemble modeling reduces uncertainty, differences among individual projections persist, reinforcing the need to interpret the results as potential trends rather than deterministic forecasts.

**Figure 9:**
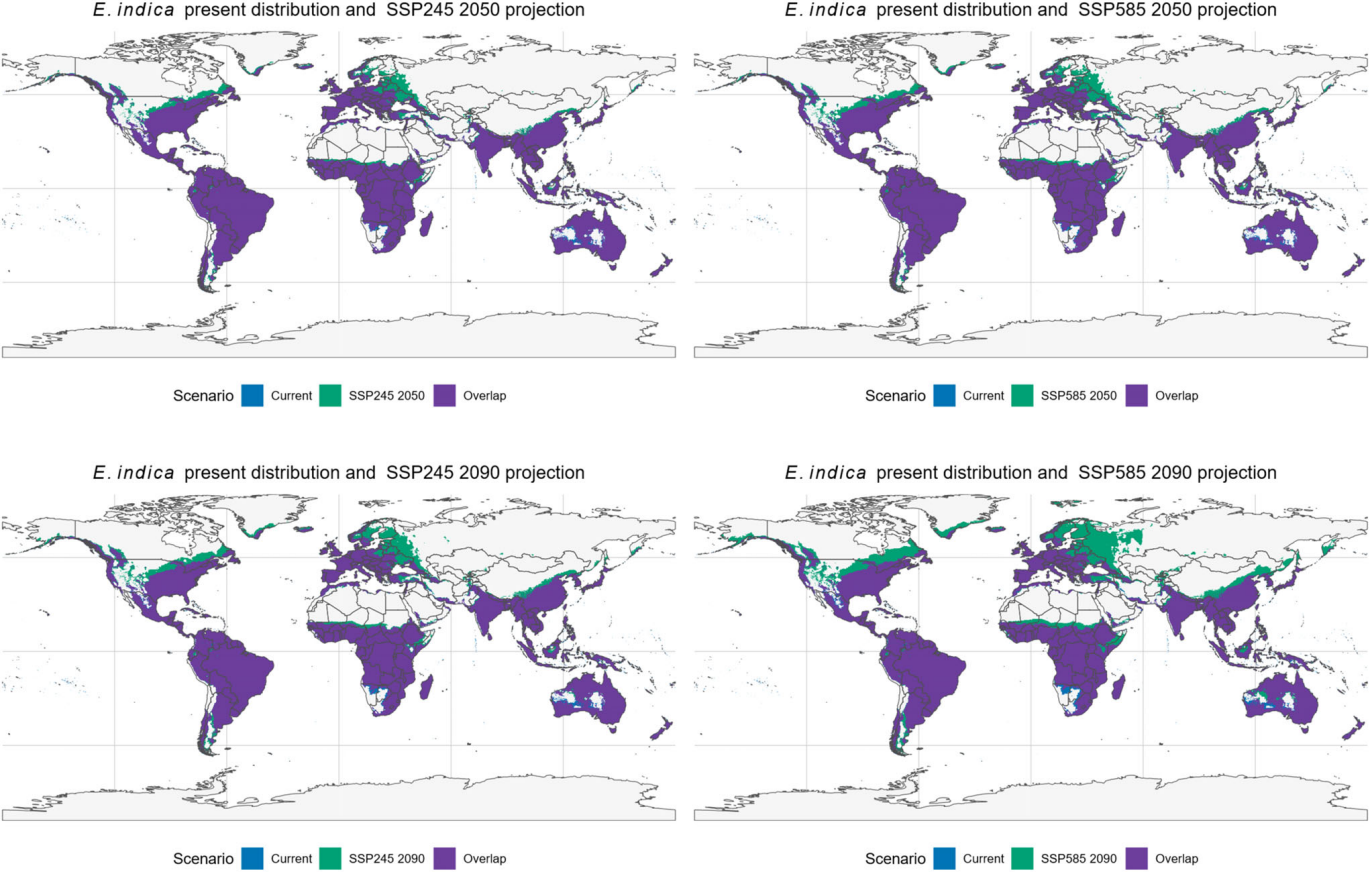
Overlap between current and future climatic suitability for *Eleusine indica* under SSP245 and SSP585 scenarios. The maps show the species' present potential distribution (blue), future projections for 2050 and 2090 under SSP245 and SSP585 (green), and areas of overlap between current and future suitability (purple). The comparison highlights stable suitable regions as well as potential range expansions under different climate scenarios.

Binary projections of climatic suitability (Fig. 9) reveal substantial stability in the potential distribution of *E. indica* between the present and future scenarios, as most currently suitable areas remain stable under SSP245 and SSP585 for both 2050 and 2090 (overlap in purple). However, an expansion into higher‐latitude regions (in green) is also evident, particularly in the Northern Hemisphere, with emphasis on North America, Europe, and Asia. This pattern becomes progressively more pronounced under SSP585 in 2090, indicating that more severe warming favors the expansion of the species' climatic niche into temperate zones. The absence of significant contraction areas suggests that climate change is more likely to expand than restrict the potential distribution of *E. indica*.

**Figure 10:**
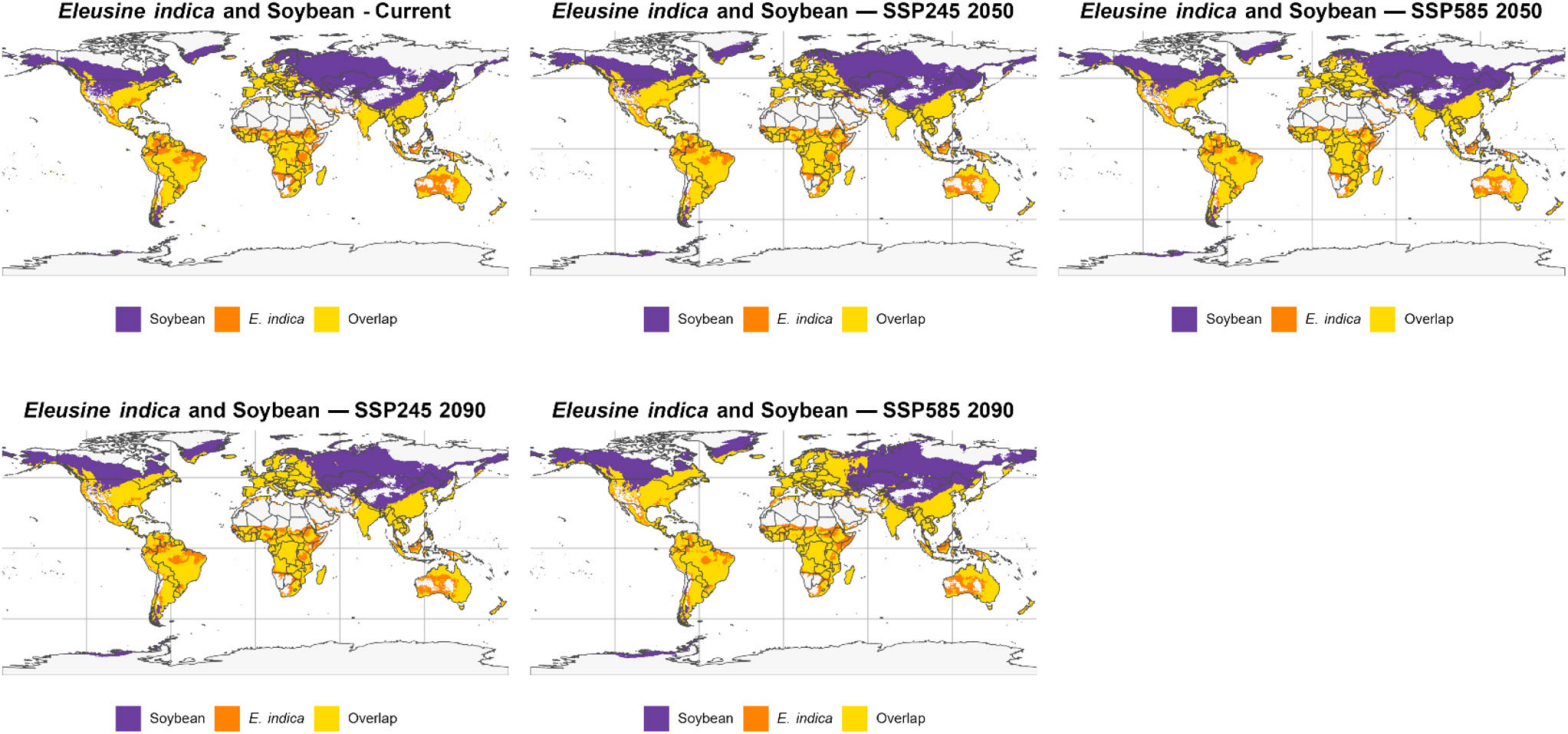
Projected overlap between *Eleusine indica* climatic suitability and soybean cultivation areas under future climate scenarios. Global climatic suitability of *E. indica* under future projections, highlighting stable, expanding, and contracting areas. Purple areas indicate contraction, orange areas expansion, and yellow areas stable suitability compared with current conditions. Overlap between soybean cultivation zones (purple) and projected suitability of *E. indica* (orange) under SSP245 and SSP585 scenarios for 2050 and 2090. Yellow areas represent regions where both soybean and *E. indica* co‐occur, illustrating critical zones of potential agronomic conflict.

**Figure 11:**
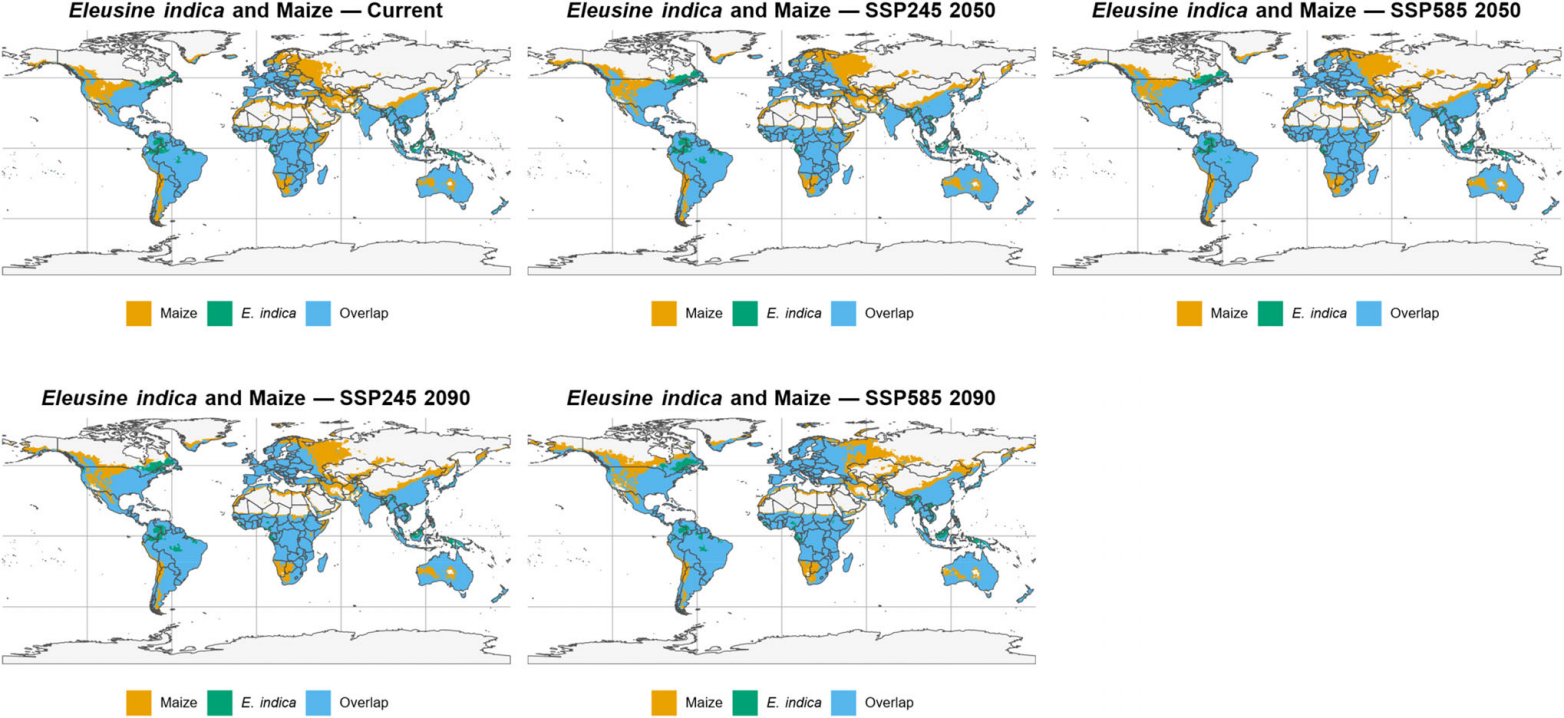
Projected overlap between *Eleusine indica* climatic suitability and maize cultivation areas under future climate scenarios. Global climatic suitability of *E. indica* under current and projected conditions, highlighting regions of stable, expanding, and contracting suitability. Orange areas represent maize cultivation zones, green areas indicate suitable regions for *E. indica*, and blue areas denote areas of overlap between both, illustrating potential zones of agronomic conflict under current and projected climatic conditions (SSP245 and SSP585) for the years 2050 and 2090.

The overlap projections between the potential distribution of *E. indica* and areas suitable for soybean and maize cultivation (Figs 10 and 11) reveal consistent spatial patterns that highlight critical zones for agricultural management at a global scale. For both crops, there is extensive spatial coincidence in strategic regions of South America (especially Brazil, Argentina, and Paraguay), Southeast Asia, and the US Corn Belt, indicating areas of high vulnerability to the species' expansion. In contrast, much of the projected overlap in higher latitudes, such as northern Europe, Russia, and Canada, occurs in areas that are currently not heavily cultivated, representing long‐term alert zones as climate change may promote agricultural expansion into these regions.

For both soybean and maize, future scenarios (SSP245 and SSP585 for 2050 and 2090) indicate the persistence or expansion of overlap areas in major global production regions, especially under high‐emission scenarios. This alignment between the potential expansion of *E. indica* and strategic agricultural hubs reinforces that the impacts are not limited to an increase in the species' climatic niche but also involve greater overlap with areas of intensive cultivation. This situation increases the risks of competition, control failures, and rising management costs.

## 4 DISCUSSION

### 4.1 Model performance and reliability

In this study, the predictive performance of the four algorithms was (AUC = 0.999; TSS/kappa = 0.980), surpassing the values expected by chance. An AUC of 0.5 indicates that predictions are no better than random, whereas higher values reflect increasing model accuracy and reliability.^12^, ^45^ Previous theoretical and empirical studies have demonstrated that presence‐only distribution models can robustly capture species–environment relationships, and that AUC values confirm their ability to discriminate suitable from unsuitable areas across a wide range of taxa and environmental contexts.^46^ The consistently reliable model performance obtained here therefore suggests that the ensemble projections are ecologically robust and meaningful, rather than statistical artifacts.

Moderate spatial autocorrelation detected in the residuals (Moran's *I* = 0.65) reflects the structured distribution of presences, which is expected for ecological data since species occurrences are rarely random in space. Although spatial autocorrelation can artificially inflate model performance, the consistently high predictive accuracy across algorithms indicates that this effect did not compromise the reliability of the results. Similar patterns have been reported in other ENM studies, where spatial dependence was recognized as a natural property of ecological processes rather than solely as a statistical bias.^47^

The positive MESS values (14.33 ± 0.97) demonstrate that model projections were carried out predominantly under environmental conditions analogous to those used during calibration, thereby minimizing the risk of extrapolation to non‐analog climates. This outcome reinforces the robustness of predictions, as models tend to lose reliability when transferred to novel environmental spaces. Similar applications of MESS in the ENM have shown its effectiveness in distinguishing areas of reliable interpolation from zones of environmental extrapolation, often revealing that a large fraction of projected areas may fall into uncertain conditions if not properly assessed.^48^ More recently, MESS analysis has also been applied to identify not only areas of low environmental similarity but also the most dissimilar variables driving habitat change under future climate scenarios,^49^ highlighting its importance for ensuring ecological realism in projections.

Regions characterized by low MOP values tend to coincide with areas of limited agricultural activity or highly heterogeneous land‐use systems, where climatic conditions differ substantially from those prevailing in major crop‐producing regions. This spatial pattern suggests that uncertainty associated with strict extrapolation is concentrated in regions of lower agronomic relevance, reducing potential bias in projections over major agricultural landscapes. Consequently, projected suitability gains in intensively cultivated regions are primarily supported by environmentally analogous conditions, strengthening the confidence in predicted invasion risks for global crop production systems.^40^

### 4.2 Current distribution and future expansion

The projections for *Eleusine indica* indicate relative stability in its global distribution under moderate climate scenarios, with only limited range contractions or expansions projected for mid‐century (2050). This pattern is consistent with the species' broad climatic tolerance, particularly its ability to persist across wide gradients of temperature and seasonal precipitation.

Under the high‐emission scenario (SSP585), however, a pronounced expansion of climatically suitable areas is projected by the end of the century, with approximately 13 million km^2^ of newly suitable areas emerging, despite concurrent contractions, resulting in a net gain of about 9 million km^2^ relative to current conditions. Such expansion is likely driven by the relaxation of thermal constraints in regions currently limited by low minimum temperatures or strong climatic seasonality, combined with shifts in precipitation regimes that enhance establishment and persistence in disturbed landscapes. Similar climate‐driven mechanisms linking temperature extremes and precipitation variability to large‐scale range expansion have been reported for invasive species under future warming scenarios.^50^ Together, these processes reinforce the ecological plasticity of *E. indica* and its capacity to colonize newly suitable environments under future climate change.

These findings align with patterns observed for other invasive grasses. For instance, the modeling of *Phragmites australis* in Iraq also revealed climate‐driven redistributions, with habitat gains in some regions and losses in others.^12^ While *P. australis* showed an overall decline in high‐suitability habitats under both moderate and high‐emission pathways, *E. indica* displayed the opposite trend, with future expansion particularly under SSP585. Such contrasts illustrate that invasive success is species‐specific, shaped by physiological tolerances, water dependence, and climatic thresholds.

From an agronomic perspective, the stability of *E. indica* under moderate scenarios is concerning, as it indicates that current management challenges are unlikely to diminish. Its projected expansion under high‐emission pathways further exacerbates risks to global agriculture, particularly in strategic production zones such as the US Corn Belt, the Indo‐Gangetic Plain, and South America. These regions are already under intense weed pressure and are projected to remain highly vulnerable, while additional climatically suitable areas are expected to emerge in temperate zones, including parts of North America, Europe, and Asia, by the end of the century. This pattern confirms that *E. indica* will persist as a major global threat regardless of emission trajectory, with higher emissions accelerating both the pace and extent of its spread. Such projections underscore the urgent need for proactive monitoring, early detection, and integrated weed management strategies to mitigate its impact on global food security.

The widespread occurrence of *E. indica* across tropical and subtropical regions, with Brazil standing out as one of the global epicenters of invasion, highlights the species' remarkable capacity to adapt and persist under contrasting agroecological conditions. *Eleusine indica* already infests more than 60% of Brazilian grain‐producing areas, with the potential to reduce yields by over 50%,^51^ and has evolved resistance to eight different herbicide modes of action,^52^ making it one of the most challenging weeds for chemical control worldwide.

Preventive measures such as systematic surveillance, early detection programs, and integrated management practices that combine crop rotation, mechanical control, and herbicide stewardship are essential to delay or mitigate the spread of *E. indica*.^53^ In regions where the species is not yet established, investments in early warning systems are far more cost‐effective than eradication efforts once populations become widespread.^54^ The distributional trends identified in this study therefore not only reveal current invasion hotspots but also highlight priority areas for international cooperation in weed management under climate change.

In this context, the long‐term sustainability of agricultural production depends on diversifying control strategies and reducing reliance on herbicides alone. Integrated weed management practices that emphasize crop diversification, cultural tactics, and proactive resistance stewardship have been consistently identified as the most effective way to mitigate the spread and impact of invasive and resistant weeds.^55^

It has been suggested that the invasive potential of *E. indica* is linked to physiological traits such as the C_4_ photosynthetic pathway, rapid germination, long seed longevity, and efficient water use, which may allow the species to thrive in stressful environments and adapt quickly to changing conditions.^56^ In addition to these physiological advantages, *E. indica* exhibits pronounced morphological and physiological divergence between biotypes from agricultural fields and turfgrass environments, reflecting rapid local adaptation to contrasting selective regimes. Agricultural biotypes show taller growth, longer leaves, and fewer tillers, traits favoring competition for light within crop canopies, whereas turfgrass biotypes display dwarfism, increased tillering, and greater reproductive allocation, indicative of adaptation to mowing and mechanical stress. These differences highlight the species' ability to adjust its life‐history strategies in response to environmental and anthropogenic pressures. Moreover, evidence of altered gibberellin metabolism and possible epigenetic modulation in dwarf phenotypes suggests that *E. indica* can exploit both genetic and non‐genetic mechanisms to persist under recurrent disturbance. Together, these observations reinforce that the broad ecological amplitude and invasive potential of *E. indica* arise from a combination of high genetic variability, phenotypic flexibility, and metabolic versatility, enabling the species to maintain fitness across distinct agroecosystems and climatic conditions.^57^

On a broader scale, the success of *E. indica* may reflect broader evolutionary patterns observed within the Poaceae family, one of the most successful plant groups in colonizing and dominating ecosystems worldwide. This success has been attributed to a combination of life‐history and ecological traits, including high phenotypic plasticity, efficient dispersal, and adaptability to disturbed habitats, rather than to a single photosynthetic type. The invasive ability of grasses is closely associated with their high genetic diversity and exposure to regional selective pressures, which promote rapid adaptation to climatic variability and anthropogenic environments such as intensively managed agricultural systems.^58^

In this context, the substantial genetic diversity of *E. indica* underpins its broad geographic distribution, the recurrent and independent emergence of herbicide‐resistant biotypes, and its ability to persist across highly contrasting agroecosystems. Phylogenetic evidence further suggests that Poaceae lineages with greater functional diversity and more recent diversification events tend to display enhanced adaptive potential.

Such evolutionary diversity provides a wide range of functional traits that allow grasses to thrive across environments and respond swiftly to selective pressures. In agricultural and natural systems, where environmental changes or selective forces such as intensive herbicide use are recurrent, this diversity may be a decisive factor driving the adaptive success of *E. indica* and other invasive grasses.^59^ However, *E. indica* deserves particular attention because it combines a cosmopolitan distribution with exceptional ecological plasticity and multiple herbicide resistance mechanisms, making it one of the few species capable of simultaneously threatening tropical, subtropical, and temperate agroecosystems. Its rapid global spread, agronomic impact, and recurrent evolution of resistance make it an emblematic model for understanding how adaptive diversity translates into large‐scale invasion success under climate change.

Biological evidence supports the modeled trends by revealing a combination of anatomical, physiological, and biochemical traits that confer broad adaptive potential to *E. indica*. The species exhibits amphistomatic leaves and Kranz‐type anatomy typical of C_4_ metabolism, optimizing CO_2_ fixation under high radiation and temperature and enhancing water use efficiency in arid environments.^60^ Both upland and lowland biotypes can survive prolonged flooding, maintaining photosynthetic activity, stomatal conductance, and seed production under hypoxic conditions.^61^

Each individual can produce over 100 000 viable seeds, which germinate across a wide temperature range (25–35 °C) and tolerate salinity and pH variation with minimal effect on viability.^56^ The seeds remain viable in the soil for several years and germinate readily at the surface, favoring establishment under direct seeding and reduced‐tillage systems. These features enable continuous recruitment and long‐term persistence in disturbed environments, consistent with the high suitability predicted for intensively managed agricultural regions.

At the metabolic level, *E. indica* synthesizes a diverse array of secondary metabolites, including phenols, flavonoids (vitexin, schaftoside, orientin), and benzofuran derivatives with antioxidant and allelopathic activity.^62^ Such compounds contribute to abiotic stress tolerance and may influence competitive interactions with crops and other weeds. The species' capacity to maintain redox balance and defend against oxidative stress under extreme conditions aligns with its persistence in environments characterized by high irradiance and temperature fluctuations.

The projected persistence of climatically suitable areas for *E. indica*, even under contrasting future scenarios, emphasizes the resilience of its ecological niche and the difficulty of relying on climate change to limit its distribution. This persistence likely reflects the species' inherent adaptive capacity and high environmental tolerance, consistent with the evolutionary patterns discussed earlier. Instead, the progressive appearance of new suitable areas in temperate latitudes highlights how climate change may expand the agricultural regions exposed to invasion. This pattern underscores that climate change acts less as a natural barrier and more as a facilitator of spread, reinforcing the need for proactive monitoring and coordinated management efforts in regions that have not yet experienced severe infestations.

The overlap between the potential distribution of *E. indica* and major cropping areas, particularly those of soybean and maize, highlights a critical vulnerability for global agriculture, especially in South America, Southeast Asia, and the US Corn Belt. In these strategic production zones, where soybean and maize already represent cornerstones of food and feed supply chains, the presence of a highly adaptable and herbicide‐resistant weed such as *E. indica* poses a direct threat to productivity and profitability. While soybean and maize served here as model crops to assess risks, the invasive potential of *E. indica* is not limited to these systems.^63^, ^64^, ^65^ Its capacity to colonize and persist under diverse agroecological conditions extends the threat to other globally important commodities, including wheat, rice, and cotton.^56^ This cross‐crop vulnerability significantly amplifies the challenge, as the weed's adaptability enables it to exploit management gaps across multiple agricultural systems, thereby increasing pressure on production sustainability.

At the same time, projected overlaps in higher latitudes, such as northern Europe, Russia, and Canada, do not correspond to current soybean or maize production hotspots but represent long‐term alert zones, given that climate change may progressively shift agricultural frontiers toward temperate regions. These findings indicate that climate change will likely promote the expansion of *E. indica* into new agroecosystems while reinforcing its dominance in areas where it is already problematic. Consequently, the risks posed by *E. indica* transcend any single crop and must be addressed as a broad agricultural challenge. Effective responses will require not only herbicide stewardship but also preventive monitoring, early detection, crop diversification, and the development of integrated weed management strategies adapted to different production contexts. Such measures are essential to mitigate the long‐term impact of *E. indica* on food security and to strengthen the resilience of global production systems under climate change.

Experimental and molecular evidence reinforce these projections by showing that *E. indica* integrates highly competitive ability with adaptive resistance mechanisms that sustain persistence under intensive management. Yield reductions exceeding 50% at moderate infestation densities in cotton highlight its efficiency in resource capture and rapid canopy dominance.^64^ The widespread and repeated use of herbicides has been further selected for populations combining multiple resistance mechanisms, including EPSPS gene amplification, amino acid substitutions, reduced herbicide translocation, and enhanced detoxification capacity.^66^ These biochemical and physiological adaptations operate alongside high phenotypic plasticity, allowing resistant biotypes to maintain photosynthetic performance, metabolic stability, and rapid regrowth after sublethal herbicide exposure. Such integration of resistance, plasticity, and reproductive vigor ensures sustained competitiveness across contrasting environments and reinforces the species' capacity to persist and expand under changing agricultural and climatic conditions.^67^

Together, these findings highlight *E. indica* as a model species for understanding weed adaptation under global change. Its persistence across contrasting climates and production systems exemplifies how physiological plasticity, resistance evolution, and agronomic intensification interact to shape invasive potential. Anticipating these dynamics through integrated modeling and biological evidence is therefore essential to guide preventive management and to safeguard agricultural sustainability in the coming decades.

## 5 CONCLUSIONS

The results of this study demonstrate that *E. indica* possesses a pronounced invasive potential driven by ecological plasticity and broad climatic adaptability. This weed is already a major agronomic threat in tropical and subtropical regions, particularly in South America, Africa, and Southeast Asia, and projections indicate that climate change will further facilitate its expansion into temperate zones, including major agricultural areas such as the US Corn Belt, the Mediterranean Basin, and eastern Asia.

The overlap between climatically suitable regions and the world's principal grain‐producing areas highlights a critical risk to global food security, since *E. indica* directly competes with staple crops such as maize and soybean. Its ability to adapt to diverse agroecosystems, combined with the recurrent evolution of herbicide resistance, underscores the need for coordinated monitoring and management efforts.

While ecological niche modeling provides robust and consistent evidence for the current and future distribution of *E. indica*, inherent limitations of occurrence‐based data and the exclusion of anthropogenic or biotic factors emphasize the need to complement predictive approaches with field‐based observations, resistance surveys, and integrated weed management practices. Ultimately, the persistence and expansion patterns revealed here confirm that *E. indica* is not only a present agronomic challenge but also an emerging global threat whose mitigation requires proactive, region‐specific, and internationally coordinated strategies.

## FUNDING INFORMATION

Coordenação de Aperfeiçoamento de Pessoal de Nível Superior (CAPES).

## CONFLICT OF INTEREST

The authors declare no conflicts of interest.

## Supporting information


**Table S1.** Bioclimatic predictors (BIO1–BIO19) from WorldClim 2.1 used for ecological niche modeling of *Eleusine indica*.
**Table S2.** Cumulative proportion of variance explained by the principal components derived from the bioclimatic predictors used in the ecological niche modeling.
**Table S3.** Loadings of bioclimatic variables on the principal components derived from the PCA.

## Data Availability

The data that support the findings of this study are openly available in Zenodo at https://doi.org/10.5281/zenodo.18302109.

## References

[1] van Dijk M , Morley T , Rau ML and Saghai Y , A meta‐analysis of projected global food demand and population at risk of hunger for the period 2010–2050. Nat Food 2:494–501 (2021). 10.1038/s43016-021-00322-9. 37117684

[2] Hubert B , Rosegrant M , Van Boekel MA and Ortiz R , The future of food: scenarios for 2050. Crop Sci 50:33–50 (2010). 10.2135/cropsci2009.09.0530.

[3] IPCC , in IPCC, 2023: Climate Change 2023: Synthesis Report. Contribution of Working Groups I, II and III to the Sixth Assessment Report of the Intergovernmental Panel on Climate Change, ed. by Arias P , Bustamante M , Elgizouli I , Flato G , Howden M , Méndez‐Vallejo C *et al*. Organizado Por, Geneva, Switzerland (2023).

[4] Derbile EK , Bonye SZ and Yiridomoh GY , Mapping vulnerability of smallholder agriculture in Africa: vulnerability assessment of food crop farming and climate change adaptation in Ghana. Environ Challenges 8:100537 (2022). 10.1016/J.ENVC.2022.100537.

[5] Mahapatra B , Walia M , Chitiprolu ARR , Bellapukonda MKR and Niranjan S , Vulnerability of agriculture to climate change increases the risk of child malnutrition: evidence from a large‐scale observational study in India. PLoS One 16:e0253637 (2021). 10.1371/JOURNAL.PONE.0253637. 34181668 PMC8238181

[6] Mutengwa CS , Mnkeni P and Aleck K , Climate‐smart agriculture and food security in southern Africa: A review of the vulnerability of smallholder agriculture and food security to climate change. Sustainability 15:2882 (2023). 10.3390/SU15042882.

[7] dos Santos EA , Fortini RM , Cardoso LCB and Zanuncio JC , Climate change in Brazilian agriculture: vulnerability and adaptation assessment. Int J Environ Sci Technol 20:10713–10730 (2023). 10.1007/S13762-022-04730-7/FIGURES/5.

[8] Crews TE , Carton W and Olsson L , Is the future of agriculture perennial? Imperatives and opportunities to reinvent agriculture by shifting from annual monocultures to perennial polycultures. Global Sustainability 1:e11 (2018). 10.1017/sus.2018.11.

[9] Beckie HJ , Herbicide resistance in plants. Plants 9:435 (2020). 10.3390/plants9040435. 32244672 PMC7238419

[10] Borlaug NE , Contributions of conventional plant breeding to food production. Science 219:689–693 (1983). 10.1126/SCIENCE.219.4585.689. 17814030

[11] Early R , Bradley BA , Dukes JS , Lawler JJ , Olden JD , Blumenthal DM *et al*., Global threats from invasive alien species in the twenty‐first century and national response capacities. Nat Commun 7:12485 (2016). 10.1038/ncomms12485. 27549569 PMC4996970

[12] Khwarahm NR , MaxEnt‐based distribution modeling of the invasive species Phragmites australis under climate change conditions in Iraq. Plants 14:768 (2025). 10.3390/PLANTS14050768. 40094769 PMC11901667

[13] Alexandratos N and Bruinsma J , World Agriculture towards 2030/2050: The 2012 Revision (2012). Available: www.fao.org/economic/esa.

[14] FAO , Crops and livestock products. Food and Agriculture Organization of the United Nations (FAO), Rome (2024).

[15] Zhang C , Johnson NA , Hall N , Tian X , Yu Q and Patterson EL , Subtelomeric 5‐enolpyruvylshikimate‐3‐phosphate synthase copy number variation confers glyphosate resistance in Eleusine indica. Nat Commun 14:4865 (2023). 10.1038/s41467-023-40407-6. 37567866 PMC10421919

[16] Duke SO , The history and current status of glyphosate. Pest Manag Sci 74:1027–1034 (2018). 10.1002/ps.4652. 28643882

[17] He S , Tian J , Ouyang Y , Liao Y , Qin Y , Bai L *et al*., Glyphosate resistance in Eleusine indica: involvement of CYP71AK44 in addition to EPSPS gene overexpression. J Agric Food Chem 72:23758–23765 (2024). 10.1021/acs.jafc.4c07765. 39377301

[18] Zhang C , Yu CJ , Yu Q , Guo WL , Zhang TJ and Tian XS , Evolution of multiple target‐site resistance mechanisms in individual plants of glyphosate‐resistant Eleusine indica from China. Pest Manag Sci 77:4810–4817 (2021). 10.1002/ps.6527. 34161662

[19] Chen J , Shan B , Li Z , Chen Q , Haiyan Y , Cui H *et al*., Unraveling the mechanisms of multiple resistance across glyphosate and glufosinate in *Eleusine indica* . Pestic Biochem Physiol 206:106181 (2024). 10.1016/J.PESTBP.2024.106181. 39672610

[20] Loddo D , Imperatore G , Milani A , Panozzo S , Farinati S , Sattin M *et al*., First report of glyphosate‐resistant biotype of *Eleusine indica* (L.) Gaertn. In Europe. Agronomy 10:1692 (2020). 10.3390/AGRONOMY10111692.

[21] Araújo MB , Anderson RP , Barbosa AM , Beale CM , Dormann CF , Early R *et al*., Standards for distribution models in biodiversity assessments. Sci Adv 5:eaat4858 (2019). 10.1126/SCIADV.AAT4858. 30746437 PMC6357756

[22] Pearson RG and Dawson TP , Predicting the impacts of climate change on the distribution of species: are bioclimate envelope models useful? Glob Ecol Biogeogr 12:361–371 (2003).

[23] Soberón J and Nakamura M , Niches and distributional areas: concepts, methods, and assumptions. Proc Natl Acad Sci USA 106:19644–19650 (2009). 10.1073/PNAS.0901637106. 19805041 PMC2780935

[24] Tian Y , Zhang Q , Huang H , Huang Y , Tao J , Zhou G *et al*., Aboveground biomass of typical invasive mangroves and its distribution patterns using UAV‐LiDAR data in a subtropical estuary: Maoling River estuary, Guangxi, China. Ecol Indic 136:108694 (2022). 10.1016/J.ECOLIND.2022.108694.

[25] Solís‐Montero L , Vega‐Polanco M , Vázquez‐Sánchez M and Suárez‐Mota ME , Ecological niche modeling of interactions in a buzz‐pollinated invasive weed. Glob Ecol Conserv 39:e02279 (2022). 10.1016/J.GECCO.2022.E02279.

[26] Zizka A , Silvestro D , Andermann T , Azevedo J , Ritter CD , Edler D *et al*., CoordinateCleaner: standardized cleaning of occurrence records from biological collection databases. Methods Ecol Evol 10:744–751 (2019). 10.1111/2041-210X.13152.

[27] Powney GD , Carvell C , Edwards M , Morris RKA , Roy HE , Woodcock BA *et al*., Widespread losses of pollinating insects in Britain. Nat Commun 10:1–6 (2019). 10.1038/S41467-019-08974-9. 30914632 PMC6435717

[28] Aiello‐Lammens ME , Boria RA , Radosavljevic A , Vilela B and Anderson RP , SpThin: an R package for spatial thinning of species occurrence records for use in ecological niche models. Ecography 38:541–545 (2015). 10.1111/ECOG.01132.

[29] Boria RA , Olson LE , Goodman SM and Anderson RP , Spatial filtering to reduce sampling bias can improve the performance of ecological niche models. Ecol Model 275:73–77 (2014). 10.1016/J.ECOLMODEL.2013.12.012.

[30] Döscher R , Acosta M , Alessandri A , Anthoni P , Arsouze T , Bergman T *et al*., The EC‐Earth3 earth system model for the coupled model Intercomparison project 6. Geosci Model Dev 15:2973–3020 (2022). 10.5194/GMD-15-2973-2022.

[31] Gutjahr O , Putrasahan D , Lohmann K , Jungclaus JH , Von Storch JS , Brüggemann N *et al*., Max Planck institute earth system model (MPI‐ESM1.2) for the high‐resolution model Intercomparison project (HighResMIP). Geosci Model Dev 12:3241–3281 (2019). 10.5194/GMD-12-3241-2019.

[32] Meinshausen M , Nicholls ZRJ , Lewis J , Gidden MJ , Vogel E , Freund M *et al*., The shared socio‐economic pathway (SSP) greenhouse gas concentrations and their extensions to 2500. Geosci Model Dev 13:3571–3605 (2020). 10.5194/GMD-13-3571-2020.

[33] de Andrade AFA , Velazco SJE and Júnior PD , ENMTML: an R package for a straightforward construction of complex ecological niche models. Environ Model Softw 125:104615 (2020). 10.1016/j.envsoft.2019.104615.

[34] Destro F , Facco P , García Muñoz S , Bezzo F and Barolo M , A hybrid framework for process monitoring: enhancing data‐driven methodologies with state and parameter estimation. J Process Control 92:333–351 (2020). 10.1016/J.JPROCONT.2020.06.002.

[35] Booth TH , Nix HA , Busby JR and Hutchinson MF , Bioclim: the first species distribution modelling package, its early applications and relevance to most current MaxEnt studies. Divers Distrib 20:1–9 (2014). 10.1111/DDI.12144.

[36] Breiman L , Random forests. Mach Learn 45:5–32 (2001). 10.1023/A:1010933404324/METRICS.

[37] Guo Q , Kelly M and Graham CH , Support vector machines for predicting distribution of sudden oak death in California. Ecol Model 182:75–90 (2005). 10.1016/J.ECOLMODEL.2004.07.012.

[38] Warren DL and Seifert SN , Ecological niche modeling in Maxent: the importance of model complexity and the performance of model selection criteria. Ecol Appl 21:335–342 (2011). 10.1890/10-1171.1. 21563566

[39] Allouche O , Tsoar A and Kadmon R , Assessing the accuracy of species distribution models: prevalence, kappa and the true skill statistic (TSS). J Appl Ecol 43:1223–1232 (2006). 10.1111/J.1365-2664.2006.01214.X.

[40] Owens HL , Campbell LP , Lynnette Dornak L , Saupe EE , Barve N , Soberón J *et al*., Constraints on interpretation of ecological niche models by limited environmental ranges on calibration areas. Ecol Model 263:10–18 (2013). 10.1016/J.ECOLMODEL.2013.04.011.

[41] Hijmans RJ , Geographic Data Analysis and Modeling [R Package Raster Version 3.6–32]. CRAN: Contributed Packages (2025).

[42] Wickham H , ggplot2. Springer International Publishing, Cham (2016).

[43] Pebesma E , GeoSPARQL (Perry and Herring, 2012), and open source libraries that empower the open source geospatial software landscape including GDAL (Warmerdam, 2008), GEOS (GEOS development team, 2017), and liblwgeom (a PostGIS component). R I Dent J 10:439–446 (2016).

[44] Auguie B and Antonov A , “gridExtra: Miscellaneous Functions for ‘Grid’ Graphics.” (2022).

[45] Raes N and Ter Steege H , A null‐model for significance testing of presence‐only species distribution models. Ecography 30:727–736 (2007). 10.1111/J.2007.0906-7590.05041.X.

[46] Phillips SJ , Anderson RP , Dudík M , Schapire RE and Blair ME , Opening the black box: an open‐source release of Maxent. Ecography 40:887–893 (2017). 10.1111/ECOG.03049.

[47] Maruyama Y , An alternative to Moran's I for spatial autocorrelation (2015). Available: https://arxiv.org/pdf/1501.06260.

[48] Guillaumot C , Artois J , Saucède T , Demoustier L , Moreau C , Eléaume M , *et al*., Broad‐scale species distribution models applied to data‐poor areas. Prog Oceanogr 175:198–207 (2019). 10.1016/J.POCEAN.2019.04.007.

[49] Wu Z , Dong H , Li L , Zhao L and Song N , Lineage‐level species distribution model to assess the impact of climate change on the habitat suitability of Boleophthalmus pectinirostris. Front Ecol Evol 12:1364822 (2024). 10.3389/FEVO.2024.1364822/BIBTEX.

[50] Radha KO and Khwarahm NR , An integrated approach to map the impact of climate change on the distributions of *Crataegus azarolus* and Crataegus monogyna in Kurdistan region, Iraq. Sustainability 14:14621 (2022). 10.3390/SU142114621.

[51] Alcántara‐De La Cruz R , Barbosa L , Da Silva X , Takano HK , Heringer L , Barcellos J *et al*., The rise of Eleusine indica as Brazil's Most troublesome weed. Agronomy 15:1759 (2025). 10.3390/AGRONOMY15081759.

[52] Heap I , The International Herbicide‐Resistant Weed Database (2025).

[53] Vilà M and Hulme PE , Impact of Biological Invasions on Ecosystem Services. Springer International Publishing, Cham (2017).

[54] Heap I , Global perspective of herbicide‐resistant weeds. Pest Manag Sci 70:1306–1315 (2014). 10.1002/PS.3696. 24302673

[55] Beckie HJ and Harker KN , Our top 10 herbicide‐resistant weed management practices. Pest Manag Sci 73:1045–1052 (2017). 10.1002/PS.4543. 28160383

[56] Chauhan BS and Johnson DE , Germination ecology of Goosegrass (Eleusine indica): an important grass weed of rainfed Rice. Weed Sci 56:699–706 (2008). 10.1614/WS-08-048.1.

[57] Patel J , Hall ND , Harris JR and McElroy JS , Morphological and metabolic differences between turfgrass and row‐crop biotypes of goosegrass (Eleusine indica). Crop Sci 63:1602–1612 (2023). 10.1002/CSC2.20933.

[58] Pertierra LR , Martínez PA , Rubalcaba JG , Richardson DM and Miguel AO‐T , Contrasting patterns in phylogenetic and biogeographic factories of invasive grasses (Poaceae) across the globe. NPJ Biodivers 2:11 (2023). 10.1038/s44185-023-00016-4. 39242679 PMC11332090

[59] Linder HP , Lehmann CER , Archibald S , Osborne CP and Richardson DM , Global grass (Poaceae) success underpinned by traits facilitating colonization, persistence and habitat transformation. Biol Rev 93:1125–1144 (2018). 10.1111/brv.12388. 29230921

[60] Rahman MM and Sultana RS , Anatomy on leaf blade of Eleusine indica L. (Gramineae): A study on Kranz grass. EBAUB Journal 3:1–8 (2021). 10.5281/ZENODO.6508018.

[61] Scherer MB , Göergen AB , Pedrollo NT , Rubert J , Dornelles SHB and Lopes SJ , Goosegrass: morphophysiological characterization under water excess conditions. Planta Daninha 37:e019180844 (2019). 10.1590/S0100-83582019370100114.

[62] Sukor NSM , Zakri ZHM , Rasol NE and Salim F , Annotation and identification of phytochemicals from Eleusine indica using high‐performance liquid chromatography tandem mass spectrometry: databases‐driven approach. Molecules 28:3111 (2023). 10.3390/MOLECULES28073111. 37049873 PMC10095617

[63] Luz JM , Fonseca LF and Duarte IN , Selectivity of pre‐emergence herbicides in potato cv. Innovator. Hortic Bras 36:223–228 (2018). 10.1590/S0102-053620180213.

[64] Ma XY , Han WW , Wei LJ , Ya JM and Ma Y , Goosegrass (Eleusine indica) density effects on cotton (Gossypium hirsutum). J Integr Agric 14:1778–1785 (2015). 10.1016/S2095-3119(15)61058-9.

[65] Souza M d F , Henckes JR , Zobiole LHS , de Oliveira RS , Braz GBP , Constantin J *et al*., Competitive response of maize against glyphosate‐resistant Digitaria insularis and Eleusine indica. Crop Prot 183:106760 (2024). 10.1016/J.CROPRO.2024.106760.

[66] Li J , Zhang Z , Lei Q , Bugao L , Jin C , Liu X *et al*., Multiple herbicide resistance in Eleusine indica from sugarcane fields in China. Pestic Biochem Physiol 182:105040 (2022). 10.1016/J.PESTBP.2022.105040. 35249648

[67] Beckie HJ , Ashworth MB and Flower KC , Herbicide Resistance Management: Recent Developments and Trends. Plants 8:161 (2019). 10.3390/PLANTS8060161. 31181770 PMC6631825

